# Invasive Prediction of Ground Glass Nodule Based on Clinical Characteristics and Radiomics Feature

**DOI:** 10.3389/fgene.2021.783391

**Published:** 2022-01-06

**Authors:** Hui Zheng, Hanfei Zhang, Shan Wang, Feng Xiao, Meiyan Liao

**Affiliations:** Zhongnan Hospital, Wuhan University, Wuhan, China

**Keywords:** ground glass nodules (GGNs), Radiomics, Adenocarcinoma, computed tomography, Diagnostic model

## Abstract

**Objective:** To explore the diagnostic value of CT radiographic images and radiomics features for invasive classification of lung adenocarcinoma manifesting as ground-glass nodules (GGNs) in computer tomography (CT).

**Methods:** A total of 312 GGNs were enrolled in this retrospective study. All GGNs were randomly divided into training set (*n* = 219) and test set (*n* = 93). Univariate and multivariate logistic regressions were used to establish a clinical model, while the minimum redundancy maximum relevance (mRMR) and least absolute shrinkage and selection operator (LASSO) algorithm were used to select the radiomics features and construct the radiomics model. A combined model was finally built by combining these two models. The performance of these models was assessed in both training and test set. A combined nomogram was developed based on the combined model and evaluated with its calibration curves and C-index.

**Results:** Diameter [odds ratio (OR), 1.159; *p* ＜ 0.001], lobulation (OR, 2.953; *p* = 0.002), and vascular changes (OR, 3.431; *p* ＜ 0.001) were retained as independent predictors of the invasive adenocarcinoma (IAC) group. Eleven radiomics features were selected by mRMR and LASSO method to established radiomics model. The clinical model and radiomics mode showed good predictive ability in both training set and test set. When two models were combined, the diagnostic area under the curve (AUC) value was higher than the single clinical or radiomics model (training set: 0.86 vs. 0.83 vs. 0.82; test set: 0.80 vs. 0.78 vs. 0.79). The constructed combined nomogram could effectively quantify the risk degree of 3 image features and Rad score with a C-index of 0.855 (95%: 0.805∼0.905).

**Conclusion:** Radiographic and radiomics features show high accuracy in the invasive diagnosis of GGNs, and their combined analysis can improve the diagnostic efficacy of IAC manifesting as GGNs. The nomogram, serving as a noninvasive and accurate predictive tool, can help judge the invasiveness of GGNs prior to surgery and assist clinicians in creating personalized treatment strategies.

## Introduction

Lung cancer is the major cancer leading in cancer-related deaths, and imaging played an important role in diagnosis and treatment. With the popularity of computed tomography (CT) and artificial intelligence (AI), the discovery of lung cancer manifesting as ground-glass nodules (GGNs) increased sequentially during the process of CT screening. Early detection, follow-up, and timely intervention were of positive significance for GGNs. No doubt, these findings deserved the attention of society, medical professionals, and the general public.

GGNs could be divided into pure ground-glass nodules (pGGNs) and mixed ground-glass nodules (mGGNs) according to the presence of the solid composition. At present, the development mechanism of GGNs was not clear. GGNs may exist in various pathological entities, including tumor, inflammation, focal hemorrhage, and focal interstitial fibrosis (Park et al., 2007). Although GGN was in nonspecific radiologic findings, persistent GGN was more likely to be malignant. Studies had shown that 20% of pGGNs and 40% of mGGNs increase gradually or show a trend of increasing solid composition (Kobayashi et al., 2018). However, the GGN growth was slow and the process of deterioration may take several years, which was why multiple current guidelines recommend longer follow-up times.

Surgical resection was the most effective method for GGN treatment. Preinvasive lesions and minimally invasive adenocarcinoma (MIA) could also be well treated by lobectomy (wedge resection or segmental resection), with a 5-year disease-free survival rate of 100%. It was necessary to analyze the imaging characteristics of each pathological subtype before operation and to judge the infiltrability of the GGN.

Earlier studies had paid more attention to GGN imaging features, such as size, consolidation, and morphological characteristics. Medical imaging technology had been developing in recent years, and its use in clinical oncology had expanded from the initial diagnostic tools to personalized treatment and management tools. Artificial intelligence and radiomics diagnosis were widely concerned. Radiomics referred to the automatic extraction of a large number of quantitative features from medical images by computer software and the use of statistical methods to screen and establish diagnosis related to the results. The radiomics model showed good sensitivity and specificity in tumor pathological type discrimination and invasive judgment.

The aim of this study was to explore the diagnostic value of imaging features and radiomics features in the invasive diagnosis of lung adenocarcinoma manifested as GGN, so as to assist clinical diagnosis and treatment.

## Materials and Methods

### Patients

This retrospective study was approved by the corresponding institutional review board (grant: 2021057), and the patients’ informed consent was waived. Clinical data and chest CT image of resected GGN between July 2017 and December 2020 at Zhongnan Hospital of Wuhan University were retrospectively collected. A total of 291 patients with 312 GGNs were enrolled in this retrospective study. The inclusion criteria were as follows: (1) the nodules showed as GGN at lung window setting (width 1500 HU; level is –700 HU), image thickness ≤1.25 mm; 2) maximum diameter of nodules measured on lung windows <30 mm; 3) accurate surgical and pathological results must be obtained. Exclusion criteria were as follows: 1) incomplete chest CT image, heavy artifacts or poor quality; 2) GGN who have no pathological results or perform only a biopsy without surgery.

### Data Flowchart

**FIGURE 1:**
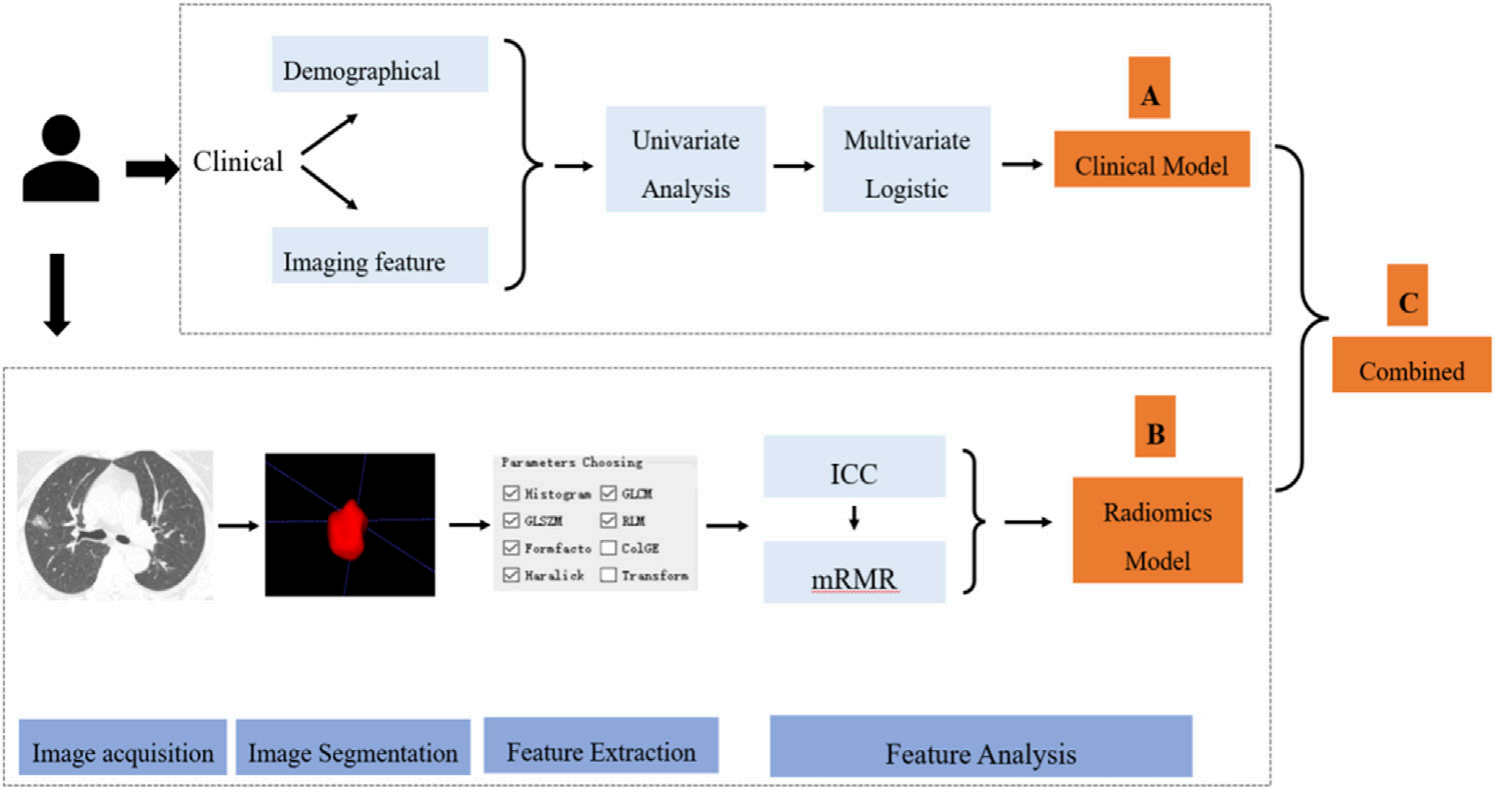
Flowchart of clinical and radiomics feature analysis.

As seen in Figure 1, the data processing of this study could be divided into three parts. The first (Figure 1A) is the clinical characteristic analysis and modeling. Clinical characteristic analysis contained univariate logistic regression and multivariate logistic regression step by step; two types of characteristics (demographics and traditional imaging features) were considered in this part. The second (Figure 1B) is the image analysis and radiomics modeling, which contained image acquisition, image segmentation, radiomics feature extraction, and modeling step by step. In this study, several most used machine learning models and a deep learning (DL) method were tried and compared, then the most suitable model was selected for radiomics modeling. After the analysis of these two parts, the screened clinical risk factors and constructed radiomics model were combined to construct the combined model and radiomics + clinical nomogram (Figure 1C).

All data sets were divided into a training set and a test set according to a 7:3 ratio using the stratified random sampling method, in which the samples were stratified according to different groups of IAC, and then randomly sampled; feature analysis and modeling were performed based on the training set, and the performance of constructed models was validated based on both training and test set.

### Clinical Characteristics Analysis and Clinical Modeling

Clinical characteristics contained two types: three demographics (patient sex, age, and operation mode) and 14 traditional imaging features, which were extracted from CT images, including diameter, volume, ratio of consolidation, mean CT value, mass, location, margin, shape, pleural indentation sign, bubble-like lucency, air bronchus sign, vascular change, speculation, and lobulation. A large number of studies (Yang et al., 2018) have confirmed that traditional imaging features play crucial roles in the diagnosis and pathological classification of GGN. The selection of these traditional imaging features was referred to these studies (Yang et al., 2018).

Diameter, mean CT value, volume, and ratio of consolidation were obtained by automatic cutting and calculation according to the Intelligent 4D Imaging System for Chest CT 6.8 (Hangzhou YITU Healthcare Technology Co., Ltd., Hangzhou, China). Mass was an important sign of tumor growth, which could reflect the change of tumor volume and the difference of cell density (Qi et al., 2020). Calculation formula Mass = $\frac{\mathrm{volume}\times 1000+\left(\mathrm{mean}\;\mathrm{CT}\;\mathrm{value}\right)}{1000}$ .

Count data were defined as follows. Location: divided into left upper lobe (LUL), left lower lobe (LLL), right upper lobe (RUL), right middle lobe (RML), and right lower lobe (RLL). Margin: a clear demarcation between the lesions and the surrounding lung parenchyma range, more than 75% of the perimeter was defined as clear, otherwise defined as blurred. Pleural indentation sign: linear or small patch between the nodules and the local pleural. Bubble-like lucency: boundary-clear air density or cavity within the nodules. Air bronchus sign: the bronchial shadow was seen in the increased density area. Vascular change: morphological changes of the vessels when passing through the GGN, such as dilatation, stiffness, correction, distortion. Spiculation: fine lines around the nodules point to the lung. Lobulation: the outline of the nodules was raised in multiple arc due to different growth speed.

Two experienced chest radiologists blinded evaluated these CT traditional imaging features independently and resolve the differences through discussion.

### Image Processing and Radiomics Modeling

Chest CT scans were performed using a GE Discovery 750HD scanner (GE Medical Systems, Milwaukee, WI, USA) and/or a SOMATOM Definition scanner (Siemens Healthineers, Forchheim, Germany), with a reconstruction slice thickness = 1.25 mm, slice interval = 1.25 mm, matrix size = 512 × 512, tube voltage = 120 kV, and tube current 100–350 mA. All images were then transmitted to the workstation and PACS for post-processing.

Before image analysis, all images were first resampled into the same sampling size (1 mm × 1 mm×1 mm) using the linear interpolation method. Then, the open-source image analysis software ITK-SNAP (Version 3.6; http://www.itksnap.org) was used for manual segmentation and radiomics analysis was applied to the CT images using in-house software (Artificial Intelligence Kit; GE Healthcare, Chicago, IL, USA). A total of 402 imaging texture features from the category of histogram, the gray-level co-occurrence matrix (GLCM), the gray-level size zone matrix (GLSZM), the gray-level run-length matrix (RLM), and shape- and size-based features were finally extracted from one single image (Table 1). The details of each radiomics features are shown in the Appendix.

**TABLE 1:** Summary of radiomics features used in this study.

Feature classes	No. of features	3 representative features
Histogram	42	FrequencySize, MaxIntensity, MeanValue,…
GLCM	144	ClusterProminence, ClusterShade, Correlation,…
GLSZM	11	SizeZoneVariability, HighIntensityEmphasis, IntensityVariability,…
RLM	180	GreyLevelNonuniformity, HighGreyLevelRunEmphasis, LongRunEmphasis,…
Formfactor	15	Compactness1, Maximum3DDiameter, Sphericity,…
Haralick	10	HaraEntroy, contrast, differenceEntropy,…
Total	402	

GLCM, gray-level co-occurrence matrix; GLSZM, gray-level size zone matrix; RLM, gray-level run-length matrix.

Another physician repeated the above segmentation and feature extraction steps for the test of feature reliability and reproducibility. The differences between the features generated by reader one and those by reader two (interobserver reliability), as well as the differences between the twice-generated features by reader 1 (intraobserver reproducibility), were all evaluated. Inter- and intraclass correlation coefficients (ICCs) were used to evaluate the agreement of feature extraction. A good agreement was reached when the ICC was greater than 0.8 in this study.

Minimum redundancy maximum relevance (mRMR) was used for feature reduction. Then, several machine learning models and a DL method (detailed in supplemental methods) were tried and compared in the radiomics modeling. The most suitable model was selected as the mathematical model of the radiomics model.

The combined model was constructed using multivariate logistic regression by combining the clinical risk factors with the radiomics model, which was used as an independent risk factor in the combined model. The radiomics + clinical nomogram transformed the combined model into a simple and visual graph, making the results of the prediction model more prominent and of higher clinical use value.

### Model Validation

The receiver operating characteristic (ROC) curve-related metrics were employed for the evaluation of model diagnostic abilities. The area under the curve (AUC) and Delong’s test were used to evaluate and compare the diagnosis abilities among different machine learning models and the DL method. Six ROC-related metrics, AUC, accuracy, sensitivity, specificity, positive predictive value (PPV), and negative predictive value (PPV) were used to assess the constructed radiomics and combined models. The relationship between nomogram-predicted probability and actual probability was evaluated by the calibration curve and C-index.

### Statistical Analysis

All statistical analyses were performed with SPSS (version 23.0, IBM) and R software (version 4.0.1, Vienna, Austria). Continuous variables with normal distribution were presented as mean ± SD and test by Student’s t test. Continuous variables with non-normal distribution were presented as median (interquartile range, IQR) and tested by Mann–Whitney *U* test. The differences of count data between two groups were analyzed by the chi-square test.

## Results

### Patients Characteristics

A total of 297 patients with 312 GGNs were included in the study; of these, 103 (33%) were male and 209 (67%) were female, and the median age was 58 (IQR: 50–65) years. There were 181 nodules in the IAC group and 131 nodules in the non-IAC group (25 benign lesions, 12 AAH, 20 AIS, 74 MIA). Detailed clinical information of patients is summarized in Table 2.

**TABLE 2:** Clinical characteristics of GGNs.

Characteristics	Number
Sex	
Male	103 (33.0%)
Female	209 (67.0%)
Age, year	58 (50–65)
Pathological subtype	
Benign	25 (8.0%)
AAH	12 (3.8%)
AIS	20 (6.4%)
MIA	74 (23.7%)
IAC	181 (58.0%)
EGFR mutation (*n* = 30)	
Mutation in exon 21	12 (40.0%)
Mutation in exon 19	10 (33.3%)
Wild type	8 (26.7%)
Preoperative position (*n* = 75)	
Pneumothorax	29 (38.6%)
Hemorrhage	32 (42.7%)
Without complications	14 (18.7%)
Interoperative biopsy (*n* = 197)	
Misdiagnosis	7 (3.6%)
Underestimate the infiltration	20 (10.1%)

AAH, atypical adenomatous hyperplasia; AIS, adenocarcinoma *in situ*; MIA, minimally invasive adenocarcinoma; IAC, invasive adenocarcinoma.

### Clinical Analysis and Modeling

In the training set, the univariate analysis showed that multiple clinical parameters were larger in IAC groups (Table 3), including diameter (17 vs. 11 mm, *p* < 0.001), volume (1,351 vs. 509 mm³, *p* < 0.001), ratio of consolidation (0.24 vs. 0.04, *p* < 0.001), mean CT value (−442 vs. −588 HU, *p* < 0.001), and mass (775 vs. 199 mg, *p* < 0.001). The IAC group had less pGGN and was easier to exhibit an irregular shape, pleural indentation sign, air bronchus sign, spiculation, lobulation, and vascular changes (*p* < 0.05).

**TABLE 3:** Univariate analysis of clinical and imaging features in the training and test sets.

Characteristic	Training set (219)		*p*	Test set (93)		*p*
	Non-IAC group (n = 86)	IAC group (*n* = 133)		Non-IAC group (*n* = 43)	IAC group (*n* = 50)	
Male	63 (71.6%)	86 (65.6%)	0.355	26 (60.5%)	34 (68.0%)	0.449
Age, year	57 (49–62)	61 (52–66)	0.009	55 (46–60)	60 (54–65)	0.027
Diameter, mm	11 (8–14)	17 (13–20)	＜0.001	11 (8–15)	17 (14–21)	＜0.001
Volume, mm³	509 (238–1,047)	1,351 (796–2,639)	＜0.001	552 (248–1,184)	1,517 (816–3,104)	＜0.001
Ratio of consolidation	0.04 (0–0.22)	0.24 (0.10–0.45)	＜0.001	0.04 (0–0.14)	0.28 (0.13–0.54)	＜0.001
Mean CT value, HU	−588 (−660–489)	−442 (−566–361)	＜0.001	−593 (−675–530)	−445 (−553–322)	＜0.001
Mass, mg	199 (104–393)	775 (322–1,352)	＜0.001	256 (101–520)	755 (420–1725)	＜0.001
Location			0.201			0.411
RUL	30 (34.1%)	51 (38.9%)		12 (27.9%)	22 (44.0%)	
RML	2 (2.3%)	10 (7.6%)		4 (9.3%)	3 (6.0%)	
RLL	20 (22.7%)	20 (15.3%)		5 (11.6%)	8 (16.0%)	
LUL	26 (29.5%)	41 (31.3%)		15 (34.9%)	11 (22.0%)	
LLL	10 (11.4%)	9 (6.9%)		7 (16.3%)	6 (12.0%)	
pGGN	47 (53.4%)	32 (24.4%)	＜0.001	24 (55.8%)	9 (18.0%)	＜0.001
Margin			0.106			0.377
Clear	30 (34.1%)	59 (45.0%)		22 (51.2%)	21 (42.0%)	
Unclear	58 (65.9%)	72 (55.0%)		21 (48.8%)	29 (58.0%)	
Shape			＜0.001			0.008
Round or oval	60 (68.2%)	54 (41.2%)		29 (67.4%)	20 (40.0%)	
Irregular	28 (31.8%)	81 (58.8%)		14 (32.6%)	30 (60.0%)	
Pleural indentation sign	27 (30.7%)	74 (56.5%)	＜0.001	15 (34.9%)	22 (44.0%)	0.371
Bubble-like lucency	21 (23.9%)	34 (26.0%)	0.727	6 (14.0%)	14 (28.0%)	0.1
Air bronchus sign	15 (17.0%)	66 (50.4%)	＜0.001	14 (32.6%)	31 (62.0%)	0.005
Spiculation	33 (37.5%)	72 (55.0%)	0.011	13 (30.2%)	28 (56.0%)	0.013
Lobulation	24 (27.3%)	89 (67.9%)	＜0.001	14 (32.6%)	27 (54.0%)	0.038
Vascular change	31 (35.2%)	97 (74.0%)	＜0.001	17 (39.5%)	37 (74.0%)	0.001

LLL, left lower lobe; LUL, left upper lobe; RLL, right lower lobe; RML, right middle lobe; RUL, right upper lobe.

The clinical model was built using multivariable logistic regression, where diameter [odds ratio (OR), 1.159; *p*＜0.001], lobulation (OR, 2.953; *p* = 0.002), and vascular changes (OR, 3.431; *p*＜0.001) were identified as independent risk factors. The AUC of the clinical model in the training set and the test set was 0.83 and 0.78, respectively.

### Comparison of Diagnosis Efficacy for Different Methods

**FIGURE 2:**
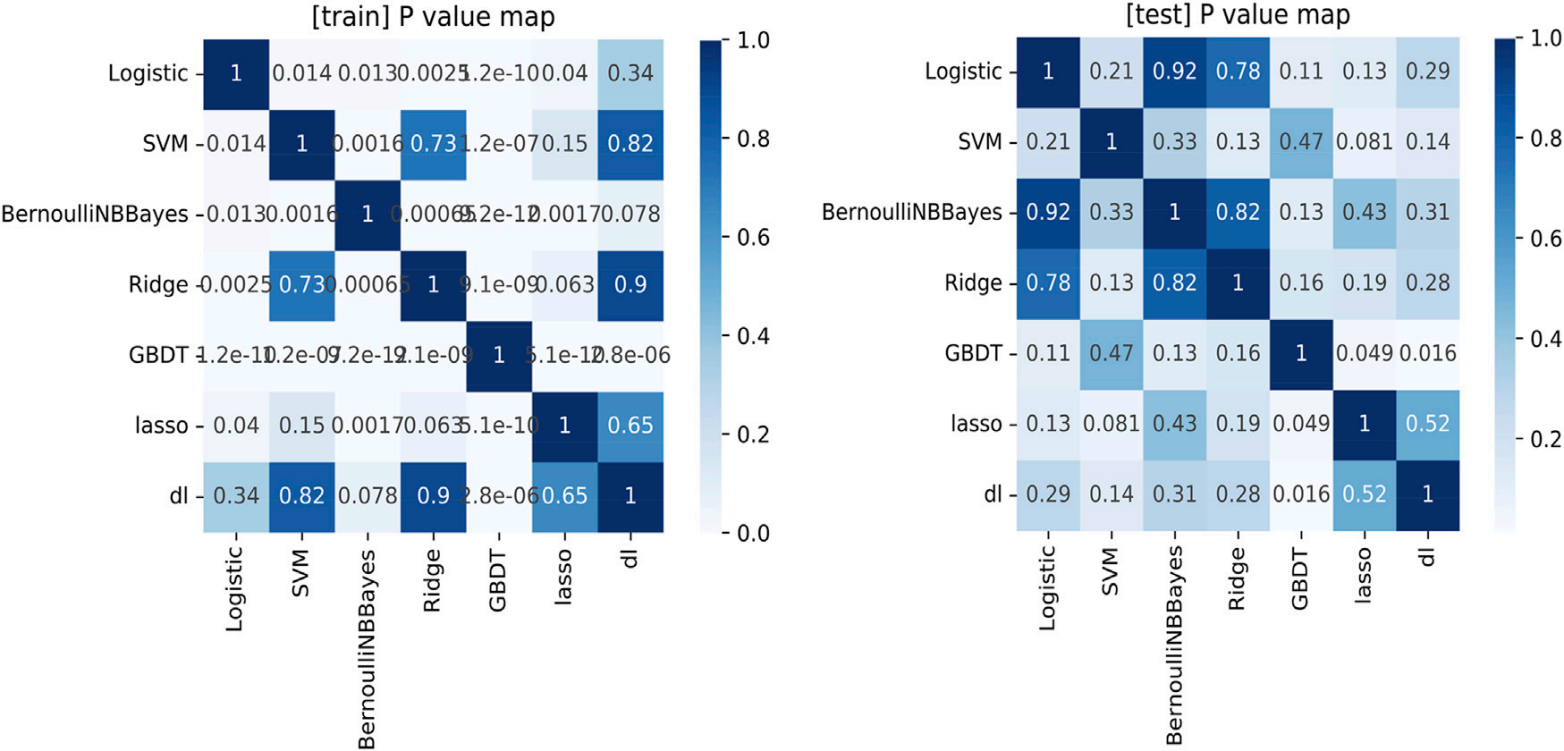
The comparison of the ROC analysis among the machine learning models in the training set and test set.

As shown in Figure 2 and Table 4, we found that for both training and test sets, DL models showed the best diagnostic performance. However, the difference between it and other models was not significant, except for the GBDT model (obvious overfitting). The diagnostic ability of LASSO was the second highest in the test set, but similarly their difference was not significant. The LASSO model was a linear regression method using L1 regularization, which could make the learned weights of some features 0, so as to achieve the purpose of feature sparseness and selection. Considering that its model structure is simple and not easy to overfit with a strong clinical interpretability, we choose LASSO as the mathematical model of the radiomics model for this study.

**TABLE 4:** Results of the ROC analysis for different machine learning methods.

	Training set			Test set		
	AUC	[0.025	0.975]	AUC	[0.025	0.975]
Logistic	0.806	0.748	0.864	0.776	0.683	0.869
SVM	0.836	0.781	0.891	0.750	0.651	0.849
Bernoulli naive Bayes	0.780	0.718	0.843	0.778	0.685	0.870
Ridge	0.833	0.780	0.887	0.773	0.678	0.867
GBDT	1.000	NaN	NaN	0.702	0.596	0.808
LASSO	0.819	0.763	0.874	0.793	0.702	0.885
DL	0.830	0.776	0.884	0.819	0.732	0.905

SVM, support vector machine; GBDT, gradient boosting decision tree; LASSO, least absolute shrinkage and selection operator; DL, deep learning.

### Radiomics Analysis and Modeling

**FIGURE 3:**
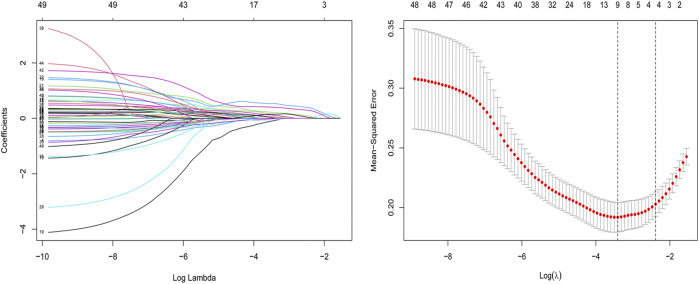
Feature selection for the LASSO logistic regression. The selection of the tuning parameter (*λ*) using a 10-fold cross-validation. At minimal value of the mean square error of the classification, the dotted vertical line (*λ* = 0.033) was drawn, including 11 optimal features with nonzero coefficients. The histogram of 11 radiomics features was presented.

After ICC analysis, 217 variables were retained and included in mRMR and LASSO analysis. Finally, 11 optimal features with nonzero coefficients were selected to establish a radiomics model (Figure 3 and Table 5). The radiomics model had AUC values of 0.82 and 0.79 in the training set and the test set, respectively.

**TABLE 5:** 11 features selected by the LASSO method.

Index	Coefficients
InverseDifferenceMoment_AllDirection_offset1_SD	0.145
ShortRunEmphasis_angle135_offset1	0.119
GLCMEnergy_angle0_offset4	−0.102
MinorAxisLength	0.218
ShortRunHighGreyLevelEmphasis_angle45_offset7	0.533
RunLengthNonuniformity_AllDirection_offset1_SD	−0.016
kurtosis	−0.053
GLCMEntropy_angle45_offset7	0.406
Percentile35	0.028
HighIntensityEmphasis	0.061
HaralickCorrelation_angle45_offset1	0.149

### Nomogram and Calibration Curve of IAC Manifested as GGNs

**FIGURE 4:**
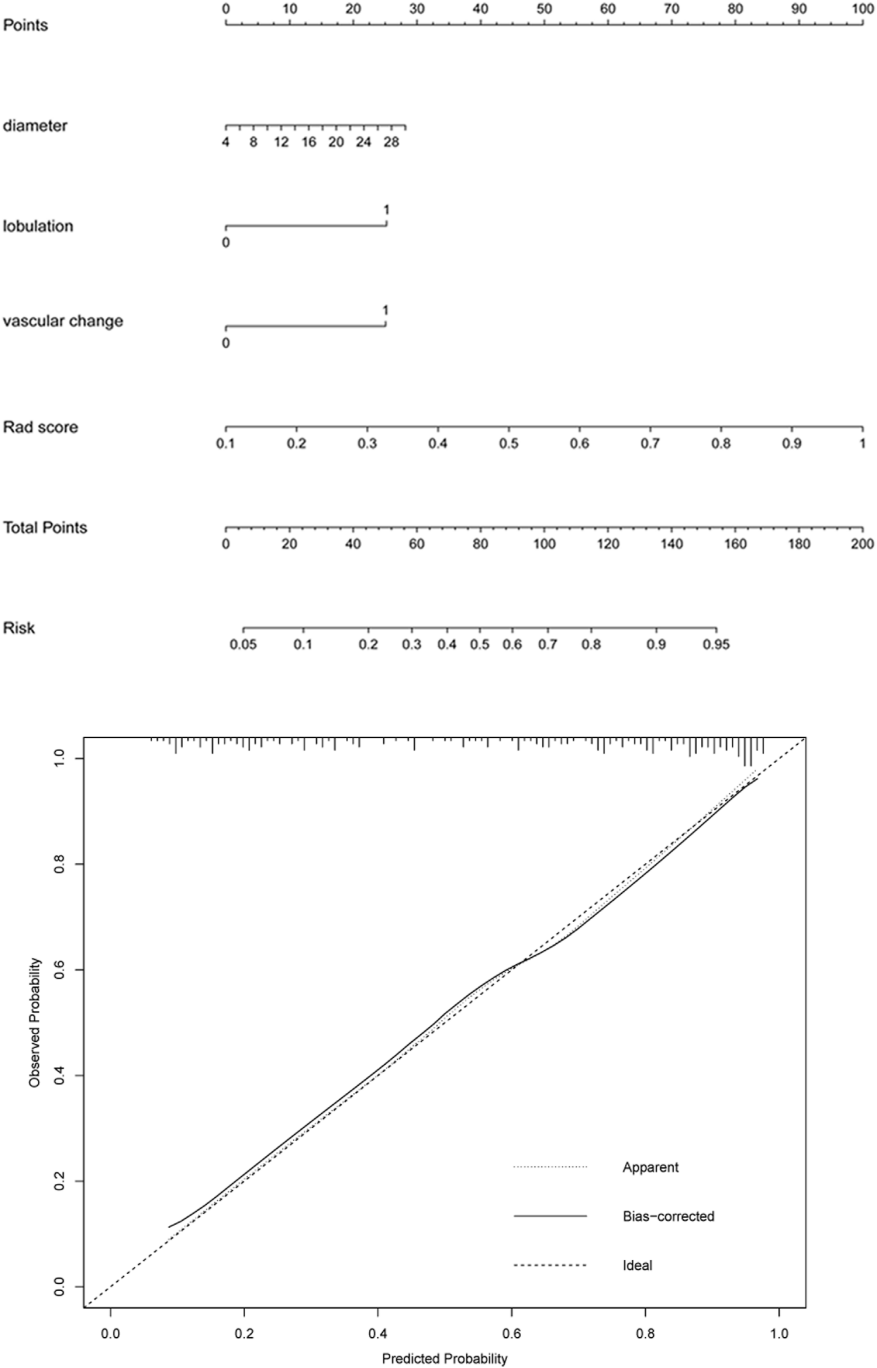
A radiomics-based nomogram was developed in the training set. The radiomics-based nomogram was developed in the training set, and the diameter, lobulation, vascular change, and Rad score were incorporated. The total score was calculated by adding the score for each risk factor, and then the probability of IAC was predicted on the risk axis.

A logistic regression analysis identified the diameter, lobulation, vascular change, and Rad score as independent predictors, which were incorporated to develop an individualized prediction nomogram (Figure 4). The calibration curve showed a high consistency between predicted probability and observed probability, and a c-index of 0.855 (95%: 0.805–0.905).

### Clinical Use of the Nomogram

**FIGURE 5:**
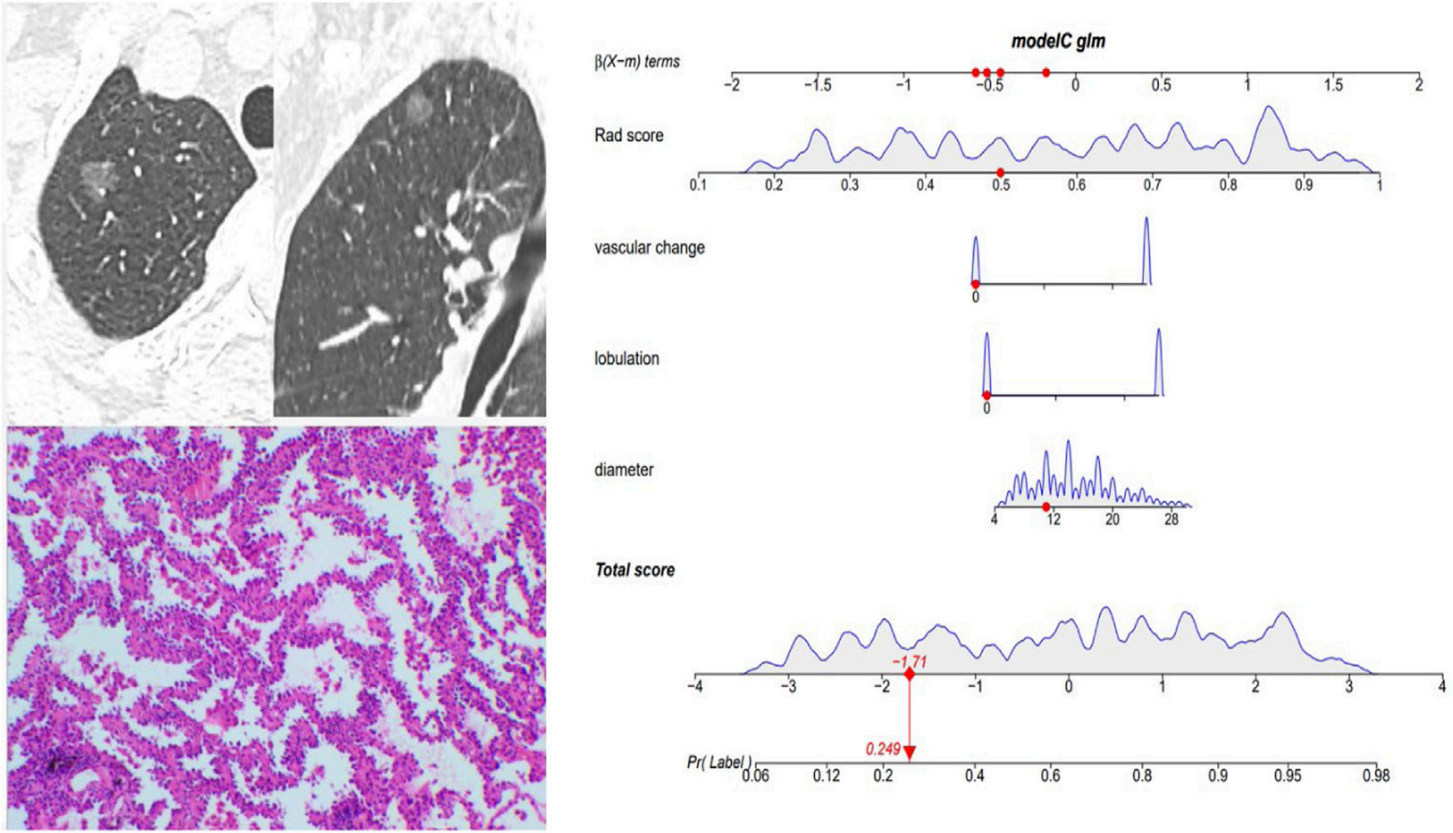
Female, 57 years old; CT showed a pGGN of 11 mm in the right upper lobe, with no significant lobulation and vascular change, and the Rad score of pGGN was 0.491. Interactive nomogram showing that the IAC risk probability of this nodule was 0.249. The case was confirmed as AAH.

**FIGURE 6:**
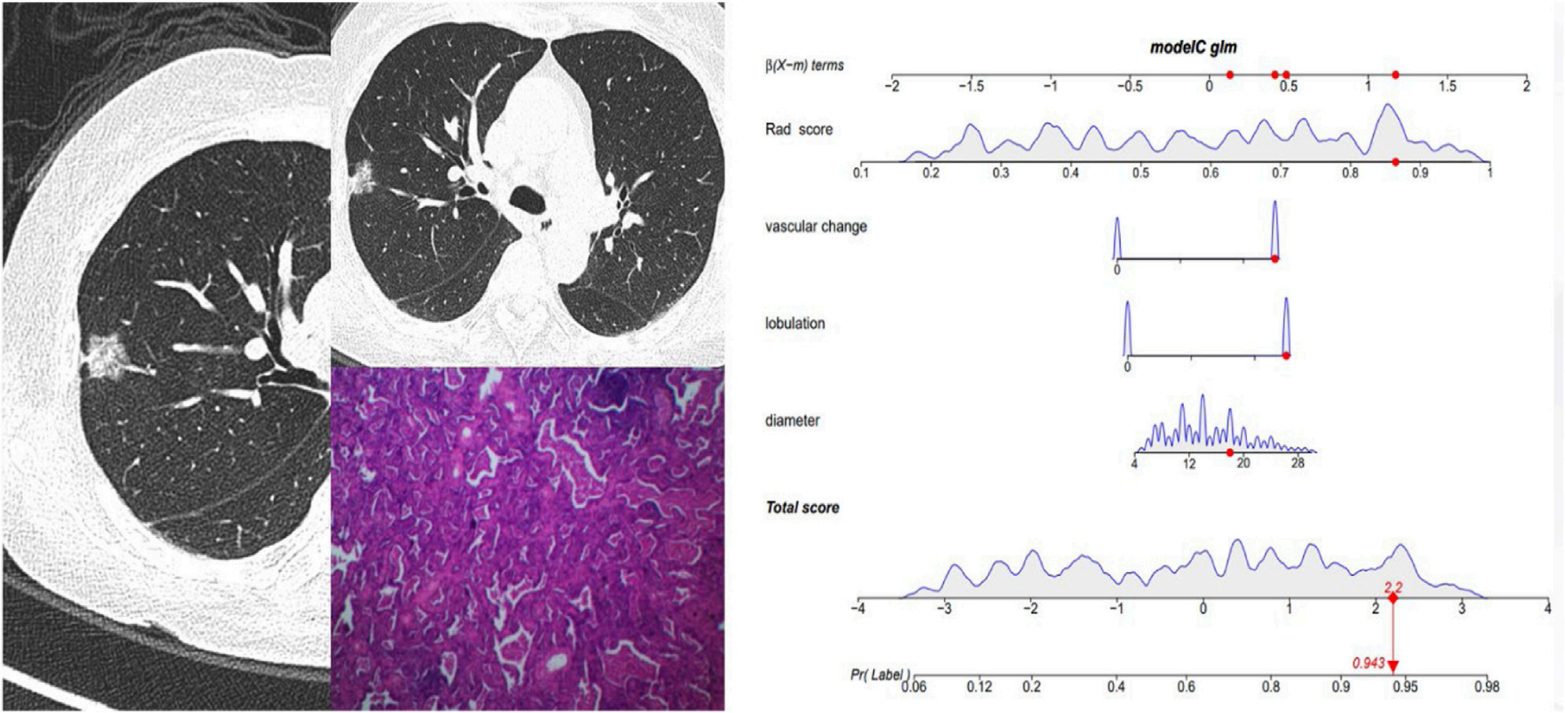
Female, 62 years old. CT showed a mGGN of 18 mm in the right upper lobe, with significant lobulation and vascular change, and the Rad score of pGGN was 0.864. The interactive nomogram showing the IAC risk probability of this nodule was 0.943. The case was confirmed as IAC.

Figure 5 and Figure 6 showed the important value of the nomogram for GGN diagnosis. The total score was calculated based on Rad score and the imaging performance of the lesion including diameter, presence of lobulation, and vascular change. Finally, the corresponding total score indicated the probability of IAC. In Figure 5, the nodule showed a low IAC risk probability of 0.249, and the final pathological was confirmed as AAH. Figure 6 showed a GGN with high IAC risk probability of 0.943, and the final pathology result was consistent with the prediction of the nomogram.

### Comparison of Diagnosis Efficiency Between Clinical Model and Radiomics Model

**FIGURE 7:**
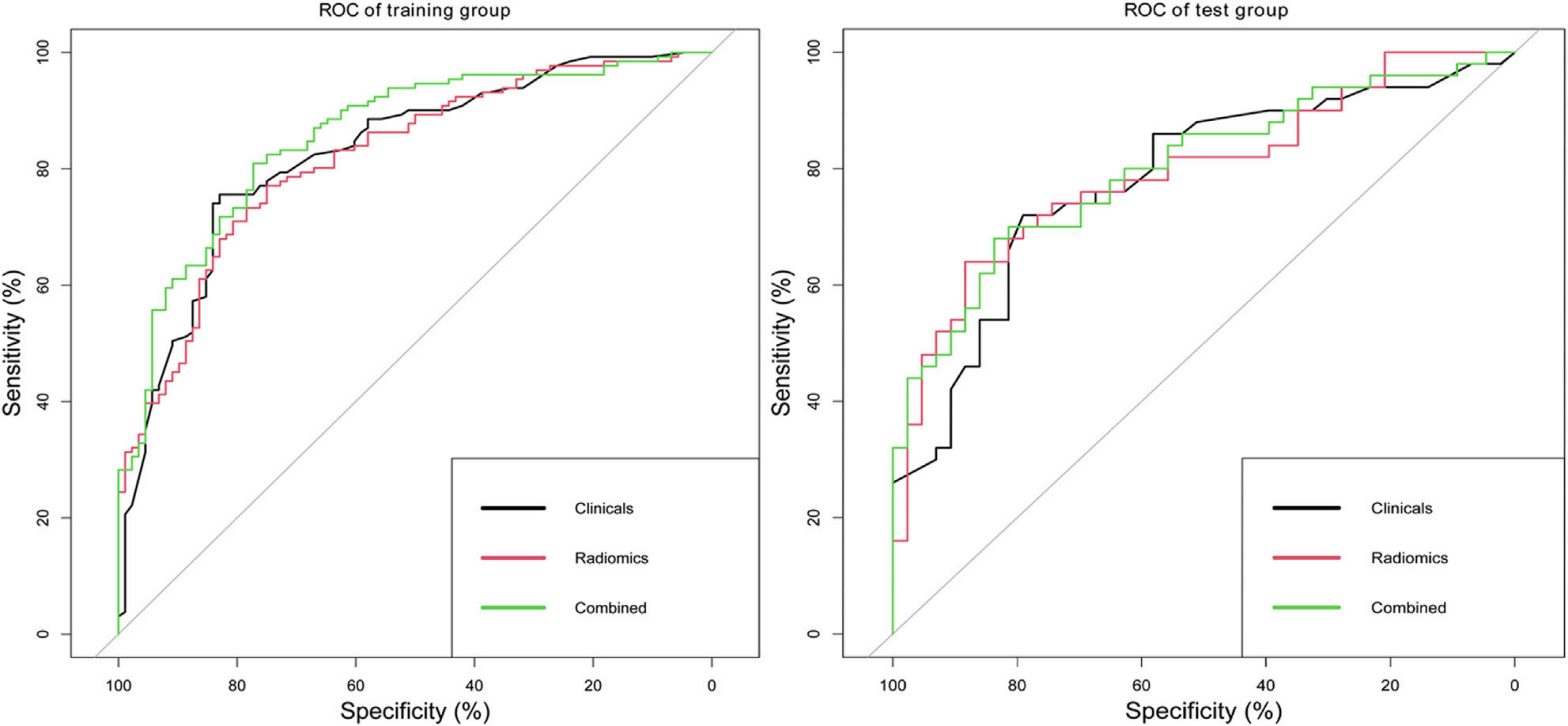
ROC analysis of clinical model, radiomics model, and combined model in the training set and test set.

Delong’s tests showed that the performance of the combined model was significantly better than that of a single clinical or radiomics model in the training set (clinical vs. combined, 0.83 vs. 0.86, *p* = 0.032; radiomics vs. combined, 0.82 vs. 0.86, *p* = 0.031). In the test set, there were no significant differences in ROC analysis for the three models. The diagnostic performances of the clinical model, radiomics model, and combined model are shown in Table 6 and Figure 7.

**TABLE 6:** Comparison of diagnosis efficiency between clinical model and radiomics model.

	AUC	Sensitivity	Specificity	PPV	NPV	Accuracy
Training set						
Clinical	0.83	0.76	0.83	0.87	0.70	0.79
Radiomics	0.82	0.77	0.75	0.82	0.69	0.76
Combined	0.86	0.81	0.77	0.84	0.73	0.79
Test set						
Clinical	0.78	0.72	0.79	0.80	0.71	0.75
Radiomics	0.79	0.64	0.88	0.86	0.68	0.75
Combined	0.80	0.68	0.84	0.83	0.69	0.75

AUC, area under the curve; PPV, positive predictive value; NPV, negative predictive value.

## Discussion

In this study, we established a clinical model and radiomics models by analyzing the imaging and radiomics characteristics of GGN and compared the diagnostic values of different models to provide a highly effective GGN diagnostic tool for the clinical diagnosis. The results showed that the diagnostic accuracy of the clinical model and the radiomics model was similar to the combined model, but the AUC value increased when the clinical and radiomics models were combined. This suggested that radiomics analysis could also be a tool for clinical diagnosis.

Not surprisingly in the selection process of different machine learning and DL models, the DL method obtained the highest diagnostic efficacy, which was due to its deep excavation of information of many high-dimensional and complex image features. Highly intelligent and automated processing data using the DL network were the mainstream direction of artificial intelligence in the future, and medical image analysis is its important application field. However, how to combine them organically is still a problem. For example, in this study, to conduct a personalized evaluation with strong clinical interpretability and high availability, we hope that the model is simple with easily understood image features. At the same time, we hope that the diagnostic efficacy of the model can be as high as possible. This is a contradiction in the modeling process, which is why we finally chose LASSO as the mathematical model.

Traditional imaging feature analysis found that diameter, lobulation, and vascular changes were independent risk factors for predicting IAC. In previous studies, several imaging characteristics were related to GGN. A meta-analysis (Dai et al., 2018) showed the limited diagnostic efficacy of single-image features of GGN, with a sensitivity range of 0.41–0.52, specificity range of 0.56–0.63, and AUC range of 0.60–0.67. Zhang et al. (Zhan et al., 2019) analyzed for GGN of 5–10 mm and found that GGNs larger than 8.12 mm and with attenuation greater than −449.52 HU were more likely to be IAC. Lobulation was another important independent risk predictor (Lee et al., 2013). Morphological changes such as lobulation justified the possibility of high invasiveness of small GGNs. Vascular changes were of important significance for the invasive judgment of GGN less than 10 mm. The IAC group was more likely to show vascular stiffness, distortion, expansion, or correction (Gao et al., 2019).

Size was a vital parameter for assessing the invasiveness of GGNs. Previous studies showed that the cutoff value of 10 mm was an optimal predictor for invasive lesions in pGGNs and 14 mm was an optimal predictor for invasive lesions in mGGNs (Lee et al., 2013). Another study showed a size difference between noninvasive and invasive group pGGN (0.74 vs. 0.90 cm, *p*＜0.001) (Sun et al., 2020).

The consolidation had the potential to identify the infiltration of the GGN. The consolidation/tumor ratio (CTR) was commonly used to assess the proportion of consolidation (Kobayashi et al., 2018). However, the ratio of consolidation in this study was not an independent risk factor of IAC, which may be related to different measurement methods. In 2013, Fleischner Society proposed that the consolidation should be evaluated in the mediastinal window and its size should be evaluated based on the average of the measured long and short diameters (Naidich et al., 2013). One study noted that the average diameter of consolidation in the mediastinum may not be the most suitable to assess mGGN progress (Kakinuma et al., 2015). Now most researchers observed and measured the consolidation of nodules on the lung window (Lee et al., 2014; Zhang et al., 2014). In addition, the size of the consolidation in the mediastinal window does not equal to the size of the infiltration focal point in the pathological specimen. Since part of the alveolar collapse, inflammatory, and fibrosis changes also appear as high density, the size of consolidation on the CT image may be larger than the actual range of pathological invasiveness.

Radiomics analysis provides a method to quantify and monitor changes in the treatment process (Aerts et al., 2014). Latest developments in image acquisition, standardization, and analysis promote an objective and accurate quantitative analysis that can be used as a non-invasive diagnostic prediction method. Zhang et al. (2019) used histogram information and morphological features to construct invasive diagnostic models, with a sensitivity and specificity of 79.4% and 91.4%, respectively. Sun et al. (2020) found that the AUC of the combined model was higher than that of a single clinical model or radiomics model (training group: 0.8 vs. 0.75 vs. 0.73; validation group: 0.77 vs. 0.71 vs. 0.72). In addition to studying the tumor’s own characteristics, radiomics can also further analyze the lung changes around the tumor by obtaining ROI in the peripheral region of nodules (Huang et al., 2018).

In terms of treatment, Ginsberg and Rubinstein (1995) had suggested that the long-term effect of lobectomy was better than sublobar resection. Recent studies have proposed sublobar resection rather than traditional lobectomy for AIS or pGGN manifesting as pGGN less than 20 mm (Watanabe et al., 2002; Yoshida et al., 2005). Surgical indications of GGN have not been uniform, and surgery is usually recommended for GGN with increased diameter or increased solid composition (Gould et al., 2013). Intraoperative freezing biopsy of early lung adenocarcinoma plays an important role in determining the surgical strategy. In this study, the diagnostic accuracy of frozen biopsy was high (benign/malignant diagnosis accuracy of 96.5%; pathological subtype diagnosis accuracy of 83.2%) and could help in diagnosis and classification and guide surgical treatment. When intraoperative frozen biopsy could not provide a timely diagnosis, radiomics may serve as a reliable reference for predicting pathological classification (Wang et al., 2020). In this study, the diagnostic accuracy of the clinical and radiomics models was lower than that of intraoperative freezing biopsy. The models still need further optimization in order to be more suitable for clinical diagnosis.

However, there are several limitations in the present study. First, this study is a retrospective research, conducted in a single center with a relatively smaller sample size. Larger sample size increases the statistical power of the diagnostic analysis which is necessary in the future, and thus a prospective cohort study should be conducted to validate these findings. More prospective data at different institutions should be analyzed to validate the clinical utility of the study results. Second, the repeatability of manual or semiautomatic tumor segmentation is an unsolved problem. Parts of GGN are close to the pleural or attached to blood vessels, which are more difficult to accurately segment and showed low repeatability (Kumar et al., 2012). Researchers propose new approaches to solve the segmentation problems of GGNs such as boundary leakage and small volume over-segmentation (Li et al., 2016). A review analysis shows that machine learning-based methods are useful for detecting and quantifying GGN (Mansoor et al., 2015). However, lung segmentation methods have not been amalgamated into single approaches or unified platforms using a single-user interface. Currently, the lung GGN segmentation is finished manually by experienced radiologists. This study will attempt to explore automated segmentation techniques to improve the efficiency of segmentation in future work.

## Conclusion

Clinical and radiomics features have high accuracy in the invasive diagnosis of GGNs. Combined analysis can improve the diagnostic efficacy of IAC manifesting as GGNs. The nomogram serves as a noninvasive and accurate predictive tool to determine the invasiveness of GGNs prior to surgery and assist clinicians in creating personalized treatment strategies.

## Data Availability

The raw data supporting the conclusion of this article will be made available by the authors, without undue reservation.
