# Supplementary material for: Invasive Prediction of Ground Glass Nodule Based on Clinical Characteristics and Radiomics Feature
Source: Front Genet. 2022 Jan 6;12:783391. doi: 10.3389/fgene.2021.783391 (PMC8770987; doi:10.3389/fgene.2021.783391)
Supplement: Supplementary file 1 [file Table1.DOCX]

**Table 1 Summary of radiomic features used in this study**

| Feature classes | No. of features | 3 representative features |
| --- | --- | --- |
| Histogram | 42 | FrequencySize, MaxIntensity, MeanValue,… |
| GLCM | 144 | ClusterProminence, ClusterShade, Correlation,… |
| GLSZM | 11 | SizeZoneVariability, HighIntensityEmphasis, IntensityVariability,… |
| RLM | 180 | GreyLevelNonuniformity, HighGreyLevelRunEmphasis, LongRunEmphasis,… |
| Formfactor | 15 | Compactness1, Maximum3DDiameter, Sphericity,… |
| Haralick | 10 | HaraEntroy, contrast, differenceEntropy,… |
| Total | 402 |  |

GLCM= the Grey level co-occurrence matrix, GLSZM =the gray level size zone matrix, RLM= the gray level Run-length matrix
