# Supplementary material for: Invasive Prediction of Ground Glass Nodule Based on Clinical Characteristics and Radiomics Feature
Source: Front Genet. 2022 Jan 6;12:783391. doi: 10.3389/fgene.2021.783391 (PMC8770987; doi:10.3389/fgene.2021.783391)
Supplement: Supplementary file 2 [file DataSheet3.DOCX]

**Fig 1. Flow chart of clinical and radiomics feature analysis**

**Fig 2. The comparison of the ROC analysis among the machine learning models in training set and test set.**

**Fig 3. Feature selection for the LASSO logistic regression.** The selection of the tuning parameter (λ) using a 10-fold cross-validation. At minimal value of the mean square error of the classification, the dotted vertical line (λ= 0.033) was drawn, including 11 optimal features with non-zero coefficients. The histogram of 11 radiomics features was presented.

**Fig 4. Radiomics-based nomogram was developed in the training set**. Radiomics-based nomogram was developed in the training set, and the diameter, lobulation, vascular change and Rad-score were incorporated. The total score was calculated by adding the score for each risk factor, and then the probability of IAC was predicted on the risk axis.

**Fig 5.** Female, 57 years old, CT showed a pGGN of 11 mm in the right upper lobe, with no significant lobulation and vascular change, Rad score of pGGN was 0.491. Interactive nomogram showing the IAC risk probability of this nodule was 0.249. Case was confirmed as AAH.

**Fig 6.** Female, 62 years old. CT showed a pGGN of 18 mm in the right upper lobe, with significant lobulation and vascular change, Rad score of pGGN was 0.864. Interactive nomogram showing the IAC risk probability of this nodule was 0.943. Case was confirmed as IAC.

**Fig 7. ROC analysis of clinical model, radiomics model and combined model in training set and test set.**
