# Supplementary material for: Invasive Prediction of Ground Glass Nodule Based on Clinical Characteristics and Radiomics Feature
Source: Front Genet. 2022 Jan 6;12:783391. doi: 10.3389/fgene.2021.783391 (PMC8770987; doi:10.3389/fgene.2021.783391)
Supplement: Supplementary file 3 [file Table2.DOCX]

**Table 2. Clinical characteristics of GGNs**

| Characteristics | Number |
| --- | --- |
| Sex |  |
| male | 103（33.0%） |
| female | 209（67.0%） |
| Age，year | 58（50~65） |
| Pathological subtype |  |
| Benign | 25（8.0%） |
| AAH | 12（3.8%） |
| AIS | 20（6.4%） |
| MIA | 74（23.7%） |
| IAC | 181（58.0%） |
| EGFR [mutation](https://fanyi.so.com/?src=onebox#genetic%20mutation) (n=30) |  |
| mutation in exon 21 | 12（40.0%） |
| mutation in exon 19 | 10（33.3%） |
| wild type | 8（26.7%） |
| Preoperative position（n=75） |  |
| pneumothorax | 29（38.6%） |
| hemorrhage | 32（42.7%） |
| without complications | 14（18.7%） |
| Interoperative biopsy（n=197） |  |
| misdiagnosis | 7（3.6%） |
| underestimate the infiltration | 20（10.1%） |

AAH= atypical adenomatous hyperplasia; AIS=adenocarcinoma in situ; MIA=minimally invasive adenocarcinoma; IAC= invasive adenocarcinoma
