# Supplementary material for: Invasive Prediction of Ground Glass Nodule Based on Clinical Characteristics and Radiomics Feature
Source: Front Genet. 2022 Jan 6;12:783391. doi: 10.3389/fgene.2021.783391 (PMC8770987; doi:10.3389/fgene.2021.783391)
Supplement: Supplementary file 4 [file Table5.DOCX]

**Table 5. 11 features selected by the LASSO method**

| **Index** | **Coefficients** |
| --- | --- |
| InverseDifferenceMoment_AllDirection_offset1_SD | 0.145 |
| ShortRunEmphasis_angle135_offset1 | 0.119 |
| GLCMEnergy_angle0_offset4 | -0.102 |
| MinorAxisLength | 0.218 |
| ShortRunHighGreyLevelEmphasis_angle45_offset7 | 0.533 |
| RunLengthNonuniformity_AllDirection_offset1_SD | -0.016 |
| kurtosis | -0.053 |
| GLCMEntropy_angle45_offset7 | 0.406 |
| Percentile35 | 0.028 |
| HighIntensityEmphasis | 0.061 |
| HaralickCorrelation_angle45_offset1 | 0.149 |
