# Supplementary material for: Invasive Prediction of Ground Glass Nodule Based on Clinical Characteristics and Radiomics Feature
Source: Front Genet. 2022 Jan 6;12:783391. doi: 10.3389/fgene.2021.783391 (PMC8770987; doi:10.3389/fgene.2021.783391)
Supplement: Supplementary file 5 [file Table3.DOCX]

**Table 3. Univariate analysis of clinical and imaging features in the training and test set**

| Characteristic | Training set(219) | | P | Test set(93) | | P |
| --- | --- | --- | --- | --- | --- | --- |
|  | non-IAC group(n=86) | IAC group(n=133) |  | non-IAC group(n=43) | IAC group(n=50) |  |
| Male（%） | 63(71.6%) | 86(65.6%) | 0.355 | 26(60.5%) | 34(68.0%) | 0.449 |
| Age，year | 57(49-62) | 61(52-66) | 0.009 | 55(46-60) | 60(54-65) | 0.027 |
| Diameter，mm | 11(8-14) | 17(13-20) | ＜0.001 | 11(8-15) | 17(14-21) | ＜0.001 |
| Volume，mm³ | 509(238-1047) | 1351(796-2639) | ＜0.001 | 552(248-1184) | 1517(816-3104) | ＜0.001 |
| Ratio of consolidation | 0.04(0-0.22) | 0.24(0.10-0.45) | ＜0.001 | 0.04(0-0.14) | 0.28(0.13-0.54) | ＜0.001 |
| Mean CT value，HU | -588(-660--489) | -442(-566--361) | ＜0.001 | -593(-675--530) | -445(-553--322) | ＜0.001 |
| Mass，mg | 199(104-393) | 775(322-1352) | ＜0.001 | 256(101-520) | 755(420-1725) | ＜0.001 |
| Location |  |  | 0.201 |  |  | 0.411 |
| RUL | 30(34.1%) | 51(38.9%) |  | 12(27.9%) | 22(44.0%) |  |
| RML | 2(2.3%) | 10(7.6%) |  | 4(9.3%) | 3(6.0%) |  |
| RLL | 20(22.7%) | 20(15.3%) |  | 5(11.6%) | 8(16.0%) |  |
| LUL | 26(29.5%) | 41(31.3%) |  | 15(34.9%) | 11(22.0%) |  |
| LLL | 10(11.4%) | 9(6.9%) |  | 7(16.3%) | 6(12.0%) |  |
| pGGN（%） | 47(53.4%) | 32(24.4%) | ＜0.001 | 24(55.8%) | 9(18.0%) | ＜0.001 |
| Margin |  |  | 0.106 |  |  | 0.377 |
| clear | 30（34.1%） | 59（45.0%） |  | 22(51.2%) | 21(42.0%) |  |
| unclear | 58（65.9%） | 72（55.0%） |  | 21(48.8%) | 29(58.0%) |  |
| Shape |  |  | ＜0.001 |  |  | 0.008 |
| round or oval | 60（68.2%） | 54(41.2%) |  | 29(67.4%) | 20(40.0%) |  |
| irregular | 28(31.8%) | 81(58.8%) |  | 14(32.6%) | 30(60.0%) |  |
| Pleural indentation sign | 27(30.7%) | 74(56.5%) | ＜0.001 | 15(34.9%) | 22(44.0%) | 0.371 |
| Bubble-like lucency | 21(23.9%) | 34(26.0%) | 0.727 | 6(14.0%) | 14(28.0%) | 0.1 |
| Air bronchus sign | 15(17.0%) | 66(50.4%) | ＜0.001 | 14(32.6%) | 31(62.0%) | 0.005 |
| Spiculation | 33(37.5%) | 72(55.0%) | 0.011 | 13(30.2%) | 28(56.0%) | 0.013 |
| Lobulation | 24(27.3%) | 89(67.9%) | ＜0.001 | 14(32.6%) | 27(54.0%) | 0.038 |
| Vascular change | 31(35.2%) | 97(74.0%) | ＜0.001 | 17(39.5%) | 37(74.0%) | 0.001 |

LLL=left lower lobe; LUL=left upper lobe; RLL=right lower lobe; RML=right middle lobe; RUL= right upper lobe.
