# Supplementary material for: Invasive Prediction of Ground Glass Nodule Based on Clinical Characteristics and Radiomics Feature
Source: Front Genet. 2022 Jan 6;12:783391. doi: 10.3389/fgene.2021.783391 (PMC8770987; doi:10.3389/fgene.2021.783391)
Supplement: Supplementary file 6 [file DataSheet2.DOCX]

**Maching learning Models**

This study tested and compared six machine learning models: Logistic Regression, Support Vector Machine (SVM), Bernoulli Naive Bayes, Ridge Regression, Gradient Boosting Decision Tree (GBDT) and Least absolute shrinkage and selection operator (LASSO). Before modeling, 30 features were screened by the mRMR method, and then the dataset was divided into the same training and test set. The six models were constructed based on these same training set and the test set, and evaluated using AUC. Delong test was used to analyze whether the AUC difference between two models was significant.

To reflect the true performance of the model, we tuned and optimized the internal hyperparameters of each model during the modeling process. Randomized search method was used in the hyperparameter optimization within a given parameter range. specifically,

(1) Logistic regression: regular parameter *penalty* and the regular coefficient *C*;

(2) SVM: kernel function *kernel* and penalty parameter *C*;

(3) Bernoulli Naive Bayes: None;

(4) Ridge: penalty coefficient *α*;

(5) GBDT: maximum tree depth *max_depth*, minimum number of samples for subdividing internal nodes *min_samples_split*, minimum number of leaf nodes *min_samples_leaf*, maximum number of features *max_feature*s and subsample ratio *subsample*;

(6) LASSO: penalty coefficient λ.

All the machine learning model construction and their hyperparameters optimization were implemented using scikit-learn package with python 3.7.0.

**Deap learning method**

The deep learning model consists of patch partition layer, four transformer stages, including 2, 2, 6, 2 transformer blocks in each stage respectively, global average pooling layer and linear layer. In patch partition layer, the 3-Channel image was split into non-overlapping patch. Each patch is set as a concatenation of the raw pixel values. In four transformer stages, several transformer blocks with multi-head self-attention layer are applied on these patches to produce hierarchical features, which is the same behaviors as the convolutional neural network such as ResNet and VGGNet. Then global average pooling layer was used to reduce dimension of extracted hierarchical features by averaging spatial feature. Finally, linear layer with softmax function generates probability of metastasis status. We implement the neural networks with Pytorch 1.4.1 and Python 3.7.0.
