# Supplementary material for: Invasive Prediction of Ground Glass Nodule Based on Clinical Characteristics and Radiomics Feature
Source: Front Genet. 2022 Jan 6;12:783391. doi: 10.3389/fgene.2021.783391 (PMC8770987; doi:10.3389/fgene.2021.783391)
Supplement: Supplementary file 7 [file Table4.DOCX]

**Table 4.** **Results of the ROC analysis for different machine learning methods**

|  | Training set | | |  | Test set | | |
| --- | --- | --- | --- | --- | --- | --- | --- |
|  | AUC | [0.025 | 0.975] |  | AUC | [0.025 | 0.975] |
| Logistic | 0.806 | 0.748 | 0.864 |  | 0.776 | 0.683 | 0.869 |
| SVM | 0.836 | 0.781 | 0.891 |  | 0.750 | 0.651 | 0.849 |
| Bernoulli Naive Bayes | 0.780 | 0.718 | 0.843 |  | 0.778 | 0.685 | 0.870 |
| Ridge | 0.833 | 0.780 | 0.887 |  | 0.773 | 0.678 | 0.867 |
| GBDT | 1.000 | NaN | NaN |  | 0.702 | 0.596 | 0.808 |
| LASSO | 0.819 | 0.763 | 0.874 |  | 0.793 | 0.702 | 0.885 |
| DL | 0.830 | 0.776 | 0.884 |  | 0.819 | 0.732 | 0.905 |

SVM = Support Vector Machine; GBDT = Gradient Boosting Decision Tree; LASSO = least absolute shrinkage and selection operator; DL= deep learning
