# Supplementary material for: Invasive Prediction of Ground Glass Nodule Based on Clinical Characteristics and Radiomics Feature
Source: Front Genet. 2022 Jan 6;12:783391. doi: 10.3389/fgene.2021.783391 (PMC8770987; doi:10.3389/fgene.2021.783391)
Supplement: Supplementary file 8 [file Table6.DOCX]

**Table 6. Comparison of diagnosis efficiency between clinical model and radiomics model**

|  | AUC | Sensitivity | Specificity | PPV | NPV | Accuracy |
| --- | --- | --- | --- | --- | --- | --- |
| Training set | |  |  |  |  |  |
| Clinicals | 0.83 | 0.76 | 0.83 | 0.87 | 0.70 | 0.79 |
| Radiomics | 0.82 | 0.77 | 0.75 | 0.82 | 0.69 | 0.76 |
| Combined | 0.86 | 0.81 | 0.77 | 0.84 | 0.73 | 0.79 |
| Test set | |  |  |  |  |  |
| Clinicals | 0.78 | 0.72 | 0.79 | 0.80 | 0.71 | 0.75 |
| Radiomics | 0.79 | 0.64 | 0.88 | 0.86 | 0.68 | 0.75 |
| Combined | 0.80 | 0.68 | 0.84 | 0.83 | 0.69 | 0.75 |

AUC=Area under the Curve; PPV= positive predictive value; NPV= negative predictive value.
