# Supplementary material for: Invasive Prediction of Ground Glass Nodule Based on Clinical Characteristics and Radiomics Feature
Source: Front Genet. 2022 Jan 6;12:783391. doi: 10.3389/fgene.2021.783391 (PMC8770987; doi:10.3389/fgene.2021.783391)

**Table 1 Summary of radiomic features used in this study**

| Feature classes | No. of features | 3 representative features |
| --- | --- | --- |
| Histogram | 42 | FrequencySize, MaxIntensity, MeanValue,… |
| GLCM | 144 | ClusterProminence, ClusterShade, Correlation,… |
| GLSZM | 11 | SizeZoneVariability, HighIntensityEmphasis, IntensityVariability,… |
| RLM | 180 | GreyLevelNonuniformity, HighGreyLevelRunEmphasis, LongRunEmphasis,… |
| Formfactor | 15 | Compactness1, Maximum3DDiameter, Sphericity,… |
| Haralick | 10 | HaraEntroy, contrast, differenceEntropy,… |
| Total | 402 |  |

GLCM= the Grey level co-occurrence matrix, GLSZM =the gray level size zone matrix, RLM= the gray level Run-length matrix

**Appendix. Image radiomics features methodology**

A region of interest (ROI) was first delineated initially around the tumor outline for the 3D ROI area. Then 402 imaging texture features from the category of histogram, the Grey level co-occurrence matrix(GLCM), the gray level size zone matrix(GLSZM), the gray level Run-length matrix(RLM), and Shape and size based features were finally extracted from one single image **Table S1.**

1. **The Grey level co-occurrence matrix (GLCM)**

$\boldsymbol{P (i, j|\theta, d)}$ represents the joint probability of certain sets of pixels having certain grey-level values. It calculates how many times a pixel with grey-level **i** occurs jointly with another pixel having a grey value **j**. By varying the displacement vector **d** between each pair of pixels.

The rotation angle of an offset: 0°,45°,90°,135° and displacement vectors (distance to the neighbor pixel: 1, 2, 3 ...), different co-occurrence distributions from the same image of reference. GLCM of an image is computed using displacement vector d defined by its radius, (distance or count to the next adjacent neighbor preferably is equal to one) and rotational angles.

1. **Energy of GLCM**

Formula

$$\sum_{i,j} {g(i,j)}^{2}$$

*g is a GLCM, Where i,j are the spatial coordinates of g (i,j).

This feature Returns the sum of squared elements in the GLCM. Range = [0 1].Energy is 1 for a constant image. Is high when image has very good homogeneity or when pixels are very similar The Property Energy is also known as uniformity, uniformity of energy, and angular second moment.

In AK Software we have **18 parameters** related to the GLCM Energy :

**GLCMEnergy_AllDirection_offset1, GLCMEnergy_AllDirection_offset1_SD,**

**GLCMEnergy_angle0_offset1, GLCMEnergy_angle45_offset1,**

**GLCMEnergy_angle90_offset1, GLCMEnergy_angle135_offset1,**

**GLCMEnergy_AllDirection_offset4, GLCMEnergy_angle0_offset4,**

**GLCMEnergy_angle45_offset4, GLCMEnergy_angle90_offset4,**

**GLCMEnergy_angle135_offset4, GLCMEnergy_AllDirection_offset4_SD,**

**GLCMEnergy_AllDirection_offset7, GLCMEnergy_angle0_offset7,**

**GLCMEnergy_angle45_offset7, GLCMEnergy_angle90_offset7,**

**GLCMEnergy_angle135_offset7, GLCMEnergy_AllDirection_offset7_SD**

1. **Entropy of GLCM**

Formula

$$-\sum_{i,j} g(i,j)\log_{2} g(i,j)$$

Entropy is a measure of randomness of intensity image.

Entropy shows the amount of information of the image that is needed for the image compression. Entropy measures the loss of information or message in a transmitted signal and also measures the image information.

In AK Software we have the **18 parameters** related to the GLCM Entropy

**GLCMEntropy_AllDirection_offset1, GLCMEntropy_AllDirection_offset1_SD,**

**GLCMEntropy_angle0_offset1, GLCMEntropy_angle45_offset1,**

**GLCMEntropy_angle90_offset1, GLCMEntropy_angle135_offset1,**

**GLCMEntropy_AllDirection_offset4, GLCMEntropy_AllDirection_offset4_SD,**

**GLCMEntropy_angle0_offset4, GLCMEntropy_angle45_offset4,**

**GLCMEntropy_angle90_offset4, GLCMEntropy_angle135_offset4,**

**GLCMEntropy_AllDirection_offset7, GLCMEntropy_AllDirection_offset7_SD,**

**GLCMEntropy_angle0_offset7, GLCMEntropy_angle45_offset7,**

**GLCMEntropy_angle90_offset7, GLCMEntropy_angle135_offset7**

1. **Inertia of GLCM**

Formula

$$\sum_{\boldsymbol{i,j}} \boldsymbol{(}\left( \boldsymbol{i-j} \right)^{\boldsymbol{2}}\boldsymbol{g(i,j))}$$

It reflects the clarity of the image and texture groove depth. The contrast is proportional to the texture groove, high values of the groove produces more clarity, in contrast small values of the groove will result in small contrast and fuzzy image.

In AK Software we have the **18 parameters** related to the Inertia.

**Inertia_AllDirection_offset1, Inertia _AllDirection_offset1_SD,**

**Inertia _angle0_offset1, Inertia _angle45_offset1,**

**Inertia _angle90_offset1, Inertia _angle135_offset1,**

**Inertia _AllDirection_offset4, Inertia _AllDirection_offset4_SD,**

**Inertia _angle0_offset4, Inertia _angle45_offset4,**

**Inertia _angle90_offset4, Inertia _angle135_offset4,**

**Inertia _AllDirection_offset7, Inertia _AllDirection_offset7_SD,**

**Inertia _angle0_offset7, Inertia _angle45_offset7,**

**Inertia _angle90_offset7, Inertia _angle135_offset7**

1. **Correlation**

Formula

$-\sum_{i,j} \frac{(i-\mu)(j-\mu)g(i,j)}{\sigma^{2}}$

Image-based Correlation measures the similarity of the grey levels in neighboring pixels, tells how correlated a pixel is to its neighbor over the whole image. Range = [-1 1]. Correlation is 1 or -1 for a perfectly positively or negatively correlated image.

In AK Software we have the **18 parameters** related to the Correlation.

**Correlation_AllDirection_offset1, Correlation _AllDirection_offset1_SD,**

**Correlation _angle0_offset1, Correlation _angle45_offset1,**

**Correlation _angle90_offset1, Correlation _angle135_offset1,**

**Correlation _AllDirection_offset4, Correlation _AllDirection_offset4_SD,**

**Correlation _angle0_offset4, Correlation _angle45_offset4,**

**Correlation _angle90_offset4, Correlation _angle135_offset4,**

**Correlation _AllDirection_offset7, Correlation _AllDirection_offset7_SD,**

**Correlation _angle0_offset7, Correlation _angle45_offset7,**

**Correlation _angle90_offset7, Correlation _angle135_offset7**

1. **Inverse Difference Moment**

Formula

$$\sum\sum\frac{1}{1+{（i-j）}^{2}}g\left( i,j \right)$$

Inverse Difference Moment (IDM) is the local homogeneity. It is high when local gray level is uniform. IDM weight value is the inverse of the Contrast weight.

In AK Software we have the **18 parameters** related to the IDM.

**InverseDifferenceMoment_AllDirection_offset1, InverseDifferenceMoment_AllDirection_offset1_SD**

**InverseDifferenceMoment_AllDirection_offset4, InverseDifferenceMoment_AllDirection_offset4_SD**

**InverseDifferenceMoment_AllDirection_offset7, InverseDifferenceMoment_AllDirection_offset7_SD**

**InverseDifferenceMoment_angle0_offset1, InverseDifferenceMoment_angle0_offset4**

**InverseDifferenceMoment_angle0_offset7, InverseDifferenceMoment_angle135_offset1**

**InverseDifferenceMoment_angle135_offset4, InverseDifferenceMoment_angle135_offset7**

**InverseDifferenceMoment_angle45_offset1, InverseDifferenceMoment_angle45_offset4**

**InverseDifferenceMoment_angle45_offset7, InverseDifferenceMoment_angle90_offset1**

**InverseDifferenceMoment_angle90_offset4, InverseDifferenceMoment_angle90_offset7**

1. **Cluster Shade**

Formula

$$\sum_{i,j} ((i-\mu)+(j-\mu))^{3}g(i,j)$$

Cluster Shade in clustered shading, we group similar view samples according to their position and, optionally, normal into clusters. In AK Software, we have the 36 parameters related to Cluster analysis, first we describe the **18 related** to the Cluster Shade.

**ClusterShade_AllDirection_offset1, ClusterShade_AllDirection_offset1_SD,**

**ClusterShade_angle0_offset1, ClusterShade_angle45_offset1,**

**ClusterShade_angle90_offset1, ClusterShade_angle135_offset1,**

**ClusterShade_AllDirection_offset4, ClusterShade_AllDirection_offset4_SD,**

**ClusterShade_angle0_offset4, ClusterShade_angle45_offset4,**

**ClusterShade_angle90_offset4, ClusterShade_angle135_offset4,**

**ClusterShade_AllDirection_offset7, ClusterShade_AllDirection_offset7_SD,**

**ClusterShade_angle0_offset7, ClusterShade_angle45_offset7,**

**ClusterShade_angle90_offset7, ClusterShade_angle135_offset7**

1. **Cluster Prominence**

Formula

$$\sum_{i,j} ((i-\mu)+(j-\mu))^{4}g(i,j)$$

Cluster Prominence is a measure of asymmetry of a given distribution, high values of this feature indicate that the symmetry of the image is low, in medical imaging low values of cluster prominence represent a smaller peak for the image grey level value and usually the grey level difference between the forms is small.

**ClusterProminence_AllDirection_offset1, ClusterProminence_AllDirection_offset1_SD,**

**ClusterProminence_angle0_offset1, ClusterProminence_angle45_offset1,**

**ClusterProminence_angle90_offset1, ClusterProminence_angle135_offset1,**

**ClusterProminence_AllDirection_offset4, ClusterProminence_AllDirection_offset4_SD,**

**ClusterProminence_angle0_offset4, ClusterProminence_angle45_offset4,**

**ClusterProminence_angle90_offset4, ClusterProminence_angle135_offset4,**

**ClusterProminence_AllDirection_offset7, ClusterProminence_AllDirection_offset7_SD,**

**ClusterProminence_angle0_offset7, ClusterProminence_angle45_offset7,**

**ClusterProminence_angle90_offset7, ClusterProminence_angle135_offset7**

1. **Haralick Correlation**

Formula

$$-\sum_{i,j} \frac{(i,j)g(i,j)-\mu_{t}^{2}}{\sigma_{t}^{2}}$$

* where $\mu_{t}$and $\sigma_{t}$are the mean and standard deviation of the row (or column, due to symmetry) sums.

**HaralickCorrelation_AllDirection_offset1, HaralickCorrelation_AllDirection_offset1_SD,**

**HaralickCorrelation_angle0_offset1, HaralickCorrelation_angle45_offset1,**

**HaralickCorrelation_angle90_offset1, HaralickCorrelation_angle135_offset1,**

**HaralickCorrelation_AllDirection_offset4, HaralickCorrelation_AllDirection_offset4_SD,**

**HaralickCorrelation_angle0_offset4, HaralickCorrelation_angle45_offset4,**

**HaralickCorrelation_angle90_offset4, HaralickCorrelation_angle135_offset4,**

**HaralickCorrelation_AllDirection_offset7, HaralickCorrelation_AllDirection_offset7_SD,**

**HaralickCorrelation_angle0_offset7, HaralickCorrelation_angle45_offset7,**

**HaralickCorrelation_angle90_offset7, HaralickCorrelation_angle135_offset7**

**2)** **Haralick texture features**

Measures the degree of similarity of the gray level of the image in the row or column direction. Represents the local grey level correlation, the greater its value, the greater the correlation;

1. **Angular Second Moment**

$$f_{1}=\sum_{i=1}^{N_{g}} \sum_{j=1}^{Ng} \left( \frac{P\left( i,j \right)}{R} \right)^{2}=\sum_{i} \sum_{j} p\left( i,j \right)^{2}$$

2. **Contrast**

The contrast feature, is a difference moment of the P matrix and is a measure of the contrast or the amount of local variations present in the image.


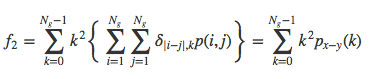


### Haralick Entropy


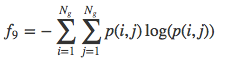


1. **HaraVariance**


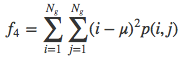


1. **sumAverage**


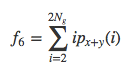


1. **sumVariance**


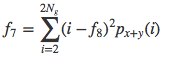


1. **sumEntropy**


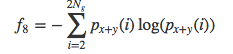


1. **differenceVariance**


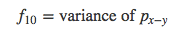


1. **differenceEntropy**


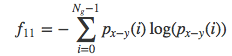


1. **inverseDifferenceMoment**


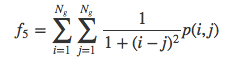


**3)** **Form Factor Parameters**

These group of features includes descriptors of the three-dimensional size and shape of the tumor region. Let in the following definitions 𝑉 denote the volume and 𝐴 the surface area of the volume of interest. We determined the following shape and size based features:

1. **Sphericity**

$$sphericity=\frac{\pi^{\frac{1}{3}}{(6V)}^{\frac{2}{3}}}{A}$$

2. **Surface area**

The surface area is calculated by triangulation (i.e. dividing the surface into connected triangles) and is defined as:

$$A=\sum_{i=1}^{N} \frac{1}{2}\left| a_{i}b_{i}\times a_{i}c_{i} \right|$$

**3. Compactness 1:**

$$compactness 1=\frac{V}{\sqrt{\pi}A^{\frac{2}{3}}}$$

**4. Compactness 2:**

$$compactness 2=36\pi\frac{V^{2}}{A^{3}}$$

**5. Maximum 3D diameter:**

The maximum three-dimensional tumor diameter is measured as the largest pairwise Euclidean distance, between voxels on the surface of the tumor volume

**6. Spherical disproportion:**

$$spherical disproportion=\frac{A}{4\pi R^{2}}$$

Where 𝑅 is the radius of a sphere with the same volume as the tumor. Where 𝑁 is the total number of triangles covering the surface and 𝑎, 𝑏 and 𝑐 are edge vectors of the triangles.

**7. Surfacetovolumeratio:**

$$surface to volume ratio=\frac{A}{V}$$

**8.VolumeCC**

The volume (V) of the tumor is determined by counting the number of pixels in the tumor region and multiplying this value by the voxel size

**9. VolumeMM**

The maximum 3D diameter, surface area and volume provide information on the size of the lesion. Measures of compactness, spherical disproportion, sphericity and the surface to volume ratio describe how spherical, rounded, or elongated the shape of the tumor is.

1. **Histogram Parameters**

First-order statistics are concerned with properties of individual pixels.

They describe the distribution of voxel intensities within the CT image through commonly used and basic metrics. Let X denote the three dimensional image matrix with N voxels and P the first order histogram divided by N_l discrete intensity levels. The following first order statistics were extracted:

1. **Energy**

$$energy=\sum_{i}^{N} {X(i)}^{2}$$

2. **Entropy**

$$entropy=-\sum_{i=1}^{N_{l}} P(i)\log_{2} P(i)$$

3. **Kurtosis**

$$kurtosis=\frac{\frac{1}{N}\sum_{i=1}^{N} {(X\left( i \right)-\bar{X})}^{4}}{\left( \sqrt{\frac{1}{N}\sum_{i=1}^{N} \left( X\left( i \right)-\bar{X} \right)^{2}} \right)^{2}}$$

where $\bar{X}$ is the mean of $X$.

1. **MaxIntensity**

The maximum intensity value of X.

1. **MeanValue**

$$mean=\frac{1}{N}\sum_{i}^{N} X(i)$$

2. **Mean absolute deviation**

The mean of the absolute deviations of all voxel intensities around the mean intensity value.

1. **MedianIntensity**

The median intensity value of $X$

1. **MinIntensity**

The minimum intensity value of $X$

1. **Range:**

The range of intensity values of $X$.

1. **Root mean square (RMS):**

$$RMS=\sqrt{\frac{\sum_{i}^{N} {X(i)}^{2}}{N}}$$

2. **Skewness:**

Represents the degree of asymmetric distribution in the image histogram. High values of Skewness means that the distribution is asymmetric otherwise the image is more symmetric; negative skew is when the numerical distribution is relatively long also called negative Skewness distribution, the opposite is referred as positive Skewness distribution (positive skew). Its possible to use The positive and negative Skewness to draw comparisons between the uniform distribution curve.

$$skewness=\frac{\frac{1}{N}\sum_{i=1}^{N} {(X\left( i \right)-\bar{X})}^{3}}{\left( \sqrt{\frac{1}{N}\sum_{i=1}^{N} {(X\left( i \right)-\bar{X})}^{2}} \right)^{3}}$$

where $\bar{X}$ is the mean of $X$.

1. **Standard deviation: stdDeviation**

$$standard deviation=\left( \frac{1}{N-1}\sum_{i=1}^{N} {(X\left( i \right)-\bar{X})}^{2} \right)^{1/2}$$

where $\bar{X}$ is the mean of $X$.

1. **Uniformity:**
$uniformity=\sum_{i=1}^{N_{l}} {P(i)}^{2}$
2. **Variance:** $variance=\frac{1}{N-1}\sum_{i=1}^{N} {(X\left( i \right)-\bar{X})}^{2}$ where $\bar{X}$ is the mean of $X$.
3. **VolumeCount**
4. **VoxelValueSum**

Represents the Sum calculations for voxels in the ROI.

1. **RelativeDeviation**

Let $\bar{X}$ denote the [mean](http://mathworld.wolfram.com/Mean.html) of a [set](http://mathworld.wolfram.com/Set.html) of quantities $X_{i}$ , then the relative deviation is defined by:

$$\frac{\Delta X_{i}}{\bar{X}}=\frac{\left| X_{i}-\bar{X} \right|}{\bar{X}}$$

1. **FrequencySize**

According to each pixel values in the GLCM.

1. **Quantiles**

Quantile normalization is a global adjustment method that assumes the statistical distribution of each sample is the same. The normalization is achieved by forcing the observed distributions to be the same and the average distribution, obtained by taking the average of each quantile across samples. They are cut points dividing the range of a probability distribution into contiguous intervals with equal probabilities, or dividing the observations in a sample in the same way.

For a finite population of *N* equally probable values indexed 1, …, *N* from lowest to highest, the *k*-th *q*-quantile of this population can equivalently be computed via the value of:

I_p_ = N k/q

In AK software, we have **5 Quantiles:**

**Quantile0.025, Quantile0.25, Quantile0.5, Quantile0.75, Quantile0.975.**

1. **Percentile**

A **percentile** (or a **centile**) is a measure used in statistics indicating the value below which a given percentage of observations in a group of observations fall.

The percentile, p%, of a distribution is defined as that value of the brightness *a* such that: P(a) = p%. or equivalently: $\int_{-\infty}^{a} P\left( \alpha\right)=p\%$

The P-th percentile $0<P\leq100$ of a list of N ordered values (sorted from least to greatest) is the smallest value in the list such that P percent of the data is less than or equal to that value. This is obtained by first calculating the ordinal rank and then taking the value from the ordered list that corresponds to that rank. The ordinal rank *n* is calculated using this formula

$$n=\frac{P}{100}*N$$

AK Software have **19 Percentiles**.

**Percentile5,** **Percentile10,** **Percentile15,** **Percentile20,** **Percentile25,** **Percentile30,** **Percentile35,** **Percentile40,** **Percentile45,** **Percentile50,** **Percentile55,** **Percentile60,** **Percentile65,** **Percentile70,** **Percentile75,** **Percentile80,** **Percentile85,** **Percentile90,** **Percentile95**

1. **Run-length matrices**

The grey level run-length matrix (RLM) P_r (i,j | θ ) is defined as the numbers of runs with pixels of gray level i and run length j for a given direction θ. RLMs is generated for each sample image segment having directions (0°,45°,90° &135°), then the following **ten statistical features** were derived: short run emphasis, long run emphasis, grey level non-uniformity, run length non-uniformity, Low Grey Level Run Emphasis, High Grey Level Run Emphasis, Short Run Low Grey Level Emphasis, Short Run High Grey Level Emphasis, Long Run Low Grey Level Emphasis and Long Run High Grey Level Emphasis.

1. **Short Run Emphasis (18 Parameters)**

Formula

$$SRE(\theta)=\frac{1}{n_{r}}\sum_{i=1}^{M} \sum_{j=1}^{N} \frac{p(i,j,\theta)}{j^{2}}$$

**ShortRunEmphasis_AllDirection_offset1, ShortRunEmphasis_AllDirection_offset1_SD,**

**ShortRunEmphasis_angle0_offset1, ShortRunEmphasis_angle45_offset1,**

**ShortRunEmphasis_angle90_offset1, ShortRunEmphasis_angle135_offset1,**

**ShortRunEmphasis_AllDirection_offset4, ShortRunEmphasis_AllDirection_offset4_SD,**

**ShortRunEmphasis_angle0_offset4,ShortRunEmphasis_angle45_offset4,**

**ShortRunEmphasis_angle90_offset4, ShortRunEmphasis_angle135_offset4,**

**ShortRunEmphasis_AllDirection_offset7,ShortRunEmphasis_AllDirection_offset7_SD,**

**ShortRunEmphasis_angle0_offset7,ShortRunEmphasis_angle45_offset7,**

**ShortRunEmphasis_angle90_offset7, ShortRunEmphasis_angle135_offset7**

1. **Long Run Emphasis (18Parameters)**

Formula

$$LRE(\theta)=\frac{1}{n_{r}}\sum_{i=1}^{M} \sum_{j=1}^{N} p(i,j,\theta)j^{2}$$

1. **Grey Level Non-uniformity(18Parameters)**

Formula

$$GLN(\theta)=\frac{1}{n_{r}}\sum_{i=1}^{M} (\sum_{j=1}^{N} p(i,j,\theta))^{2}$$

1. **Run Length Non-uniformity(18Parameters)**

Formula

$$RLN(\theta)=\frac{1}{n_{r}}\sum_{j=i}^{N} (\sum_{i=1}^{M} p(i,j,\theta))^{2}$$

1. **Low Grey Level Run Emphasis(18Parameters)**

Formula

$$LGRE(\theta)=\frac{1}{n_{r}}\sum_{j=i}^{N} \sum_{i=1}^{M} \frac{p(i,j,\theta)}{i^{2}}$$

1. **High Grey Level Run Emphasis(18Parameters)**

Formula

$$HGRE(\theta)=\frac{1}{n_{r}}\sum_{j=i}^{N} \sum_{i=1}^{M} p(i,j,\theta)i^{2}$$

1. **Short Run Low Grey Level Emphasis(18Parameters)**

Formula

$$SRLGE(\theta)=\frac{1}{n_{r}}\sum_{j=i}^{N} \sum_{i=1}^{M} \frac{p(i,j,\theta)}{i^{2}j^{2}}$$

1. **Short Run High Grey Level Emphasis(18Parameters)**

Formula

$$SRHGE(\theta)=\frac{1}{n_{r}}\sum_{j=i}^{N} \sum_{i=1}^{M} \frac{p(i,j,\theta)i^{2}}{j^{2}}$$

1. **Long Run Low Grey Level Emphasis(18Parameters)**

Formula

$$LRLGE(\theta)=\frac{1}{n_{r}}\sum_{j=i}^{N} \sum_{i=1}^{M} \frac{p(i,j,\theta)j^{2}}{i^{2}}$$

1. **Long Run High Grey Level Emphasis(18Parameters)**

Formula

$$LRHGE(\theta)=\frac{1}{n_{r}}\sum_{j=i}^{N} \sum_{i=1}^{M} p(i,j,\theta)i^{2}j^{2}$$

where $n_{r}$ is the total number of runs and $n_{p}$ is the number of pixels in the image.

1. **The gray level size zone matrix(GLSZM)**

A GLSZM describes the amount of homogeneous connected areas within the volume, of a certain size and intensity, thereby describing tumor heterogeneity at a regional scale. A voxel is considered connected if the distance is 1 according to the infinity norm. In a GLSZM P(I,j)th element equals the number of zones with gray level I and size j appear in image.

Ng be the number of discreet intensity values in the image

Ns be the number of discreet zone sizes in the image

Np be the number of voxels in the image

Nz be the number of zones in the ROI, which is equal to ∑^Ng^_i=1_∑^Ns^_j=1_P(i,j) and 1≤Nz≤Np

P(i,j) be the size zone matrix

p(i,j) be the normalized size zone matrix, defined as p(i,j)=P(i,j)/Nz

1. **SizeZoneVariability**

Formula


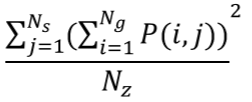


1. **HighIntensityEmphasis**

Formula


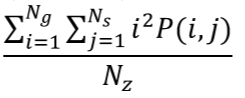


1. **HighIntensityLargeAreaEmphasis**

Formula


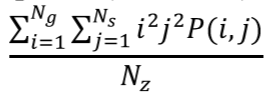


1. **HighIntensitySmallAreaEmphasis**

Formula


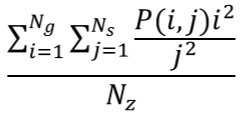


1. **IntensityVariability**

Formula


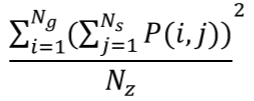


1. **LargeAreaEmphasis**

Formula


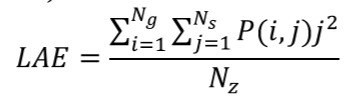


1. **LowIntensityEmphasis**

Formula


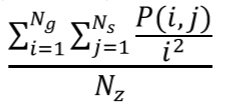


1. **LowIntensityLargeAreaEmphasis**

Formula


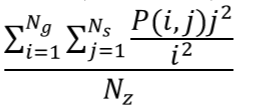


1. **LowIntensitySmallAreaEmphasis**

Formula


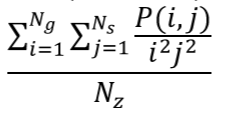


1. **SmallAreaEmphasis**

Formula


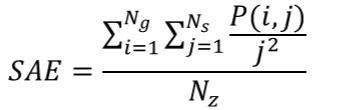


1. **ZonePercentage**

ZP measures the coarseness of the texture by taking the ratio of number of zones and number of voxels in the ROI.

Formula


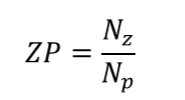

Supplement: Supplementary file 9 [file DataSheet1.DOCX]
